# Can ARNI prevent atrial fibrillation in heart failure with reduced ejection fraction? Real-world evidence meets the randomized benchmark

**DOI:** 10.1093/ehjcvp/pvag080

**Published:** 2026-09-22

**Authors:** Enrico Volpe, Stanislovas Jankauskas, Pasquale Mone, Shivangi Pande, Maria Laura Danza, Gaetano Santulli

**Affiliations:** Department of Molecular, Cellular, and Biomedical Sciences, City University of New York, School of Medicine, Manhattan, NY 10031, USA; Department of Medicine and Health Sciences, University of Molise, Campobasso 86100, Italy; Department of Molecular, Cellular, and Biomedical Sciences, City University of New York, School of Medicine, Manhattan, NY 10031, USA; Department of Medicine and Health Sciences, University of Molise, Campobasso 86100, Italy; Clinica Montevergine, GVM Care and Research, Mercogliano (Avellino) 83013, Italy; Department of Molecular, Cellular, and Biomedical Sciences, City University of New York, School of Medicine, Manhattan, NY 10031, USA; Department of Molecular, Cellular, and Biomedical Sciences, City University of New York, School of Medicine, Manhattan, NY 10031, USA; Department of Medicine and Health Sciences, University of Molise, Campobasso 86100, Italy; Department of Molecular, Cellular, and Biomedical Sciences, City University of New York, School of Medicine, Manhattan, NY 10031, USA

**This editorial refers to ‘Switching to angiotensin receptor–neprilysin inhibitor and reduced risk of new-onset atrial fibrillation in heart failure with reduced ejection fraction: a nationwide propensity score–matched study’, by Y.J. Park *et al*., https://doi.org/10.1093/ehjcvp/pvag074.**

Atrial fibrillation (AF) and heart failure (HF), among the most prevalent cardiovascular conditions of our time, share overlapping pathophysiological mechanisms, including neurohormonal activation, structural remodelling, and haemodynamic stress, which perpetuate each other in a vicious cycle.^1^ The introduction of angiotensin receptor-neprilysin inhibitor (ARNI), combining sacubitril and valsartan, has transformed HF with reduced ejection fraction (HFrEF) management. In the PARADIGM-HF trial, sacubitril/valsartan reduced the composite of cardiovascular death or HF hospitalization by 20% compared with enalapril.^2^ Beyond haemodynamic benefits, ARNI can augment natriuretic peptide signalling through neprilysin inhibition, which may lower left atrial pressure and attenuate atrial fibrosis and dilatation, providing a mechanistic rationale for AF prevention that traditional renin–angiotensin system (RAS) blockade lacks (*Figure 1*).

**Figure 1:**
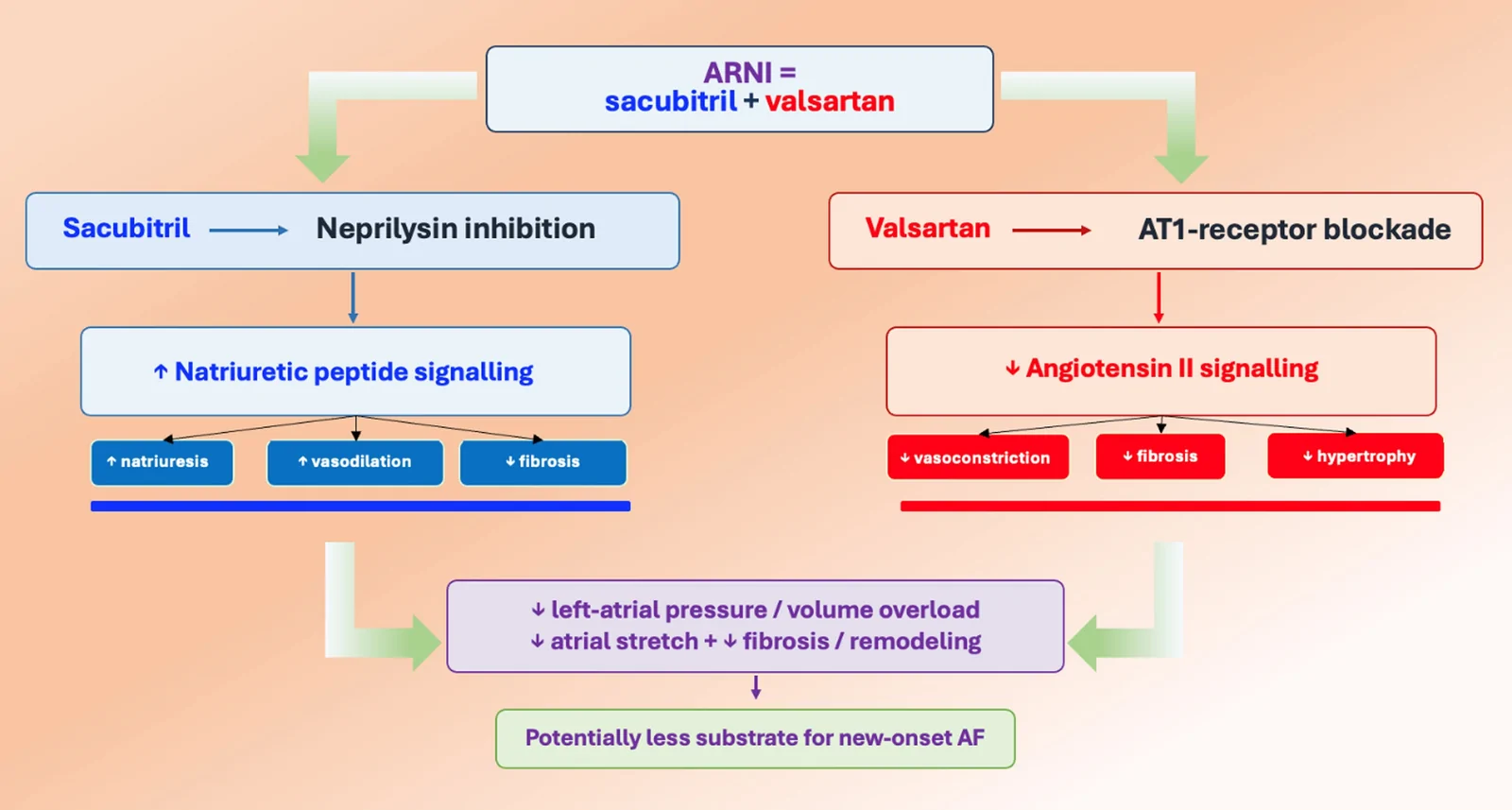
Mechanisms linking angiotensin receptor-neprilysin inhibitor to atrial remodelling.

The concept of atrial cardiomyopathy^3^ provides a framework for understanding how ARNI's effects might translate into AF prevention. Neprilysin inhibition increases the bioavailability of endogenous natriuretic peptides, which promote natriuresis, vasodilation, and reduction of pre/after-load, limiting atrial stretch. Natriuretic peptides also exert anti-fibrotic and anti-hypertrophic properties through cGMP signalling, which may attenuate the structural remodelling of atrial tissue that constitutes the substrate for AF;^4^ sacubitril/valsartan induces left atrial reverse remodelling in HFrEF patients, reducing left atrial volume index.^5^

Despite this mechanistic rationale, the randomized evidence for an AF-preventive effect of ARNI has been notably absent. In PARADIGM-HF, new-onset AF was a secondary endpoint, but the comparison yielded only 84 vs. 83 events (*P* = 0.84). Meta-analyses of randomized controlled trials have similarly failed to demonstrate significant AF reduction.^6^

This tension between a strong mechanistic rationale and null randomized evidence creates a critical evidence gap that the study by Park and colleagues, published in this issue of *European Heart Journal–Cardiovascular Pharmacotherapy*, attempts to address. The investigators examined whether switching from traditional RAS blockade to ARNI is associated with a lower risk of new-onset AF in a large cohort of HFrEF patients without prior AF. The study leverages the strengths of a comprehensive single-payer claims database, including near-complete population coverage and longitudinal pharmaceutical dispensing records, to generate real-world evidence at a scale that randomized trials have not achieved for this question.^7^ The AHA has recognized the complexity of managing AF in HFrEF, issuing a scientific statement that underscores the gaps in evidence for pharmacologic AF prevention in this population,^8^ and a recent European consensus has similarly called for more robust evidence on pharmacotherapy for HFrEF patients with concomitant AF.^9^

Switching to ARNI was associated with an 11% reduction in new-onset AF at 1-year compared with continuing RAS blockade (*P* = 0.021) in a propensity score-matched cohort of 9030 pairs. The composite outcome of new-onset AF, ischaemic stroke, or all-cause death was also reduced (*P* < 0.001). One of the greatest strengths of the study is its scale: the cohort of over 39 000 patients dwarfs the AF event counts in any single randomized trial of ARNI, and the investigators employed multiple complementary statistical approaches.

The divergence between these observational findings and the null results of PARADIGM-HF and subsequent meta-analyses demands scrutiny. The AF-naive HFrEF cohort in this study accrued far more incident AF events (805 vs. 880 in the matched cohort) than PARADIGM-HF. However, observational treatment comparisons may remain vulnerable to residual confounding.^10^ In the present study, the ARNI group was younger, more likely to be male, and more intensively treated with guideline-directed medical therapy before matching. Although propensity score matching balanced these measured covariates, the claims-based dataset lacks critical prognostic variables, including left ventricular ejection fraction, renal function, and echocardiographic measures of left atrial size. Left atrial volume is one of the most powerful predictors of incident AF, and its absence from the model represents a key unmeasured confounder. Patients selected for ARNI switching may have had less advanced atrial remodelling or better ventricular function, a difference that claims-based matching cannot capture. The differential estimated glomerular filtration rate (eGFR) thresholds for ARNI vs. angiotensin-converting enzyme (ACE) inhibitors or angiotensin receptor blockers (ARBs) further complicate the comparison.

The ‘healthy switcher’ bias deserves emphasis. By design, the ARNI group comprised patients who tolerated prior RAS blockade for a mean duration exceeding 3 years, had no documented AF over more than 3 years of prior observation, and survived at least 90 days of continuous therapy. This identifies a low-risk, treatment-adherent population, and the 1-year mortality of 6.2% confirms a favourable phenotype. Whether the observed AF association generalizes to RAS-naive, newly diagnosed, or sicker patients remains uncertain.

The claims-based AF ascertainment is another limitation. AF was defined as at least one inpatient claim or at least two outpatient claims of ICD-10 code-I48, a definition that captures neither symptom status nor monitoring intensity. Asymptomatic AF is common in HFrEF, and the true incidence is likely substantially higher than what claims data capture. If ARNI recipients were monitored more closely, ascertainment bias could inflate AF detection in that group, working against the observed association. Conversely, if sicker patients in the RAS blockade group were hospitalized more frequently, the bias could operate in the opposite direction.

The near-absence of sodium-glucose cotransporter 2 (SGLT2) inhibitors during the study period (2017–2021) is a critical limitation. SGLT2 inhibitors are a foundational therapy for HFrEF and have themselves been associated with AF reduction.^11,12^ In the current era, where SGLT2 inhibitors are widely prescribed alongside ARNI, isolating the AF-preventive contribution of ARNI from that of SGLT2 inhibitors will require careful adjustments.

The finding that beta-blocker use was associated with lower new-onset AF risk warrants cautious interpretation. A landmark individual-patient data meta-analysis found that beta-blockers did not reduce mortality in patients with HF and concomitant AF.^13^ The association observed here may reflect that beta-blocker recipients represent a less advanced disease phenotype, or that beta-blockers suppress symptomatic triggers for AF detection, rather than a true anti-arrhythmic effect on the atrial substrate.

The meta-analytic landscape for ARNI and arrhythmias provides additional context: ARNI significantly reduced the composite of sudden cardiac death and ventricular arrhythmias but showed no significant reduction in atrial arrhythmias.^14,15^ The consistency of null atrial arrhythmia findings across randomized meta-analyses stands in tension with the positive signal from this observational study.

In conclusion, Park and colleagues have conducted a rigorous observational study that generates a plausible and biologically grounded hypothesis: ARNI may reduce the risk of new-onset AF in HFrEF patients without prior AF. The main strengths include its nationwide scale, and multiple analytical approaches. However, the findings should be interpreted as hypothesis-generating. The residual confounding from unmeasured atrial structure and ventricular function, the healthy-switcher selection bias, the limitations of claims-based AF ascertainment, the absence of SGLT2 inhibitor use in the contemporary context, and the persistent null signal from randomized trials all counsel against drawing firm causal conclusions. The DAPA-HF trial demonstrated that dapagliflozin reduced worsening HF or cardiovascular death in HFrEF, and the emerging AF-preventive signal with SGLT2 inhibitors further complicates attributing AF prevention to any single drug class.^16^ What is needed now is a prospective randomized trial designed specifically to test whether ARNI prevents AF. Until such evidence is available, the AF-preventive potential of ARNI should be regarded as a promising hypothesis, and the primary rationale for ARNI use in HFrEF should remain its established benefits on mortality/hospitalization.

## Data Availability

Not applicable.
